# Intravenous plus intraventricular tigecycline-amikacin therapy for the treatment of carbapenem-resistant *Klebsiella pneumoniae* ventriculitis: A case report

**DOI:** 10.1097/MD.0000000000029635

**Published:** 2022-07-29

**Authors:** Jiyao Li, Yiguo Liu, Guangtao Wu, Hongyan Wang, Xiaoyan Xu

**Affiliations:** Department of Pharmacy, Liaocheng People’s Hospital, Liaocheng, China.

**Keywords:** Carbapenem-resistant *Klebsiella pneumoniae*, tigecycline, amikacin, intraventricular, central nervous system infections

## Abstract

**Rationale:**

Central nervous system infections (CNSIs) are one of the most serious complications after neurosurgery, especially carbapenem-resistant bacterial meningitis. Owing to the poor blood-brain barrier permeability of most antibiotics, the treatment of CNSIs by intraventricular (IVT) administration is becoming a hot topic in clinical research. Currently, the treatment of CNSIs caused by carbapenem-resistant *Klebsiella pneumoniae* is mainly based on intraventricular injection of an antibiotic combined with one or more other systemic intravenous (IV) antibiotics, whereas there are few case reports of intraventricular injection of 2 antibiotics.

**Patient concerns:**

A 57-year-old man with an open craniocerebral injury presented with dyspnea, high fever, and seizures associated with surgery.

**Diagnosis:**

Intracranial infection caused by carbapenem-resistant *K. pneumoniae* was diagnosed.

**Interventions:**

On the advice of a clinical pharmacist, the patient was given tigecycline (100 mg IV + 3 mg IVT q12h) combined with amikacin (0.8 g IV + 30 mg IVT qd) antiinfective therapy. Ultimately, the pathogens in the cerebrospinal fluid were eradicated after 7 days, and the CNSIs were completely cured after 14 days.

**Outcomes:**

The patient recovered and was discharged from the hospital without adverse reactions.

**Lessons:**

A series of in vitro and in vivo synergy tests of carbapenem-resistant *K. pneumoniae* showed that tigecycline combined with aminoglycosides had good synergistic effects and effectively suppressed bacterial resistance selection. Intravenous plus intraventricular tigecycline-amikacin seems to be a safe and effective treatment option for carbapenem-resistant *K. pneumoniae* CNSIs.

## 1. Introduction

Central nervous system infections (CNSIs) caused by gram-negative bacteria are one of the most serious complications of craniocerebral surgery and significantly affect the prognosis.^[1]^ In recent years, multidrug-resistant Enterobacteriaceae, especially *K. pneumoniae*, have emerged with the increasing use of broad-spectrum antibacterial agents. To make matters worse, the carbapenem-resistant *K. pneumoniae* (CRKP) is prevalent in patients with nosocomial infections in the United States, China, Colombia, Brazil, Argentina and a few European countries.^[2]^ According to data from the China Antimicrobial Surveillance Network (CHINET), the resistance rates of *K. pneumoniae* to meropenem and imipenem rapidly increased from 2.9 and 3.0% in 2005 to 27.1 and 25.5% in 2021, respectively. The treatment of CNSIs caused by CRKP is a difficult problem for clinicians, for the following reasons:^[2,3]^ (1) *K. pneumoniae* carbapenemase can effectively hydrolyze most β-lactams including carbapenems; (2) CRKP is generally only susceptible to a few antibiotics, including colistin, tigecycline, ceftazidime/avibactam and one or more aminoglycosides; (3) it is difficult for most drugs to reach the minimal inhibitory concentration (MIC) in the cerebrospinal fluid (CSF) because of the low penetration of the blood-brain barrier. To overcome these disadvantages, many cases and clinical studies have attempted to treat CNSIs caused by multidrug-resistant bacteria using intraventricular (IVT) injections of antibiotics to increase drug concentrations in the CSF. Here, we describe a case of CRKP intracranial infection after neurosurgery, that was successfully treated with intravenous (IV) plus IVT tigecycline-amikacin therapy with the participation of clinical pharmacists.

## 2. Case Report

A 57-year-old man underwent hematoma removal from an epidural and subdural and skull depression fracture repair plus debridement suture because of an open craniocerebral injury. An epidural drain was inserted. After the operation, vancomycin 1g q12h IV was administered empirically due to severe contamination from a head skin laceration. On hospital day 8, the patient presented with dyspnea, high fever (peak at 39.5℃) and seizures associated. A computed tomography scan of the chest and head showed obvious exudative lesions in both lower lungs with atelectasis, multiple cerebral contusions and laceration, paranasal sinus effusion, and local edema in left frontal lobe. The patient was diagnosed with pneumonia and a highly suspected intracranial infection. On hospital day 9, the white blood cell count (WBC) increased to 30.29 × 10^9^/L, C-reactive protein was 200 mg/L and procalcitonin (PCT) was 2.26 ng/mL. Lumbar CSF showed white blood cell count was 885 × 10^6^/L, leukocyte multinucleate ratio (LMR) was 97.7%, protein was 281.7mg/dL, glucose was 0.17 mmol/L and the simultaneous blood glucose level was 14.68 mmol/L (Table 1). In the meantime, Preliminary Gram staining showed gram-negative rods in the CSF and sputum. Empirical IV meropenem 2 g q8h and cefoperazone-sulbactam sodium(2:1) 3 g q8h were used for the treatment of pneumonia and intracranial infections. On hospital day 10, sputum culture revealed CRKP which was only sensitive to tigecycline, amikacin and gentamicin (Table 2). After consultation with clinical pharmacists and considering the patient history of CSF leakage, it was believed that the pathogenic bacteria of the lung and intracranial infection were homologous. With the informed consent of the patient’s family, an right extraventricular drain was placed and the antibiotics were adjusted to tigecycline (100 mg IV + 3 mg IVT q12h) + amikacin (0.8 g IV + 30 mg IVT qd). IVT antibiotics were administered as follows: 100 mg tigecycline was dissolved in 100 mL normal saline and 3 ml diluted solution was slowly injected through the extraventricular drain. Subsequently, 30 mg amikacin was injected in the same manner. Finally, the ventricular drain was closed for 2 hours to prevent early antibiotic outflow. On hospital day 11, CSF culture also indicated CRKP. After 14 days of dual IVT therapy, the CSF culture from hospital day 17 was negative and the levels of white blood cells, glucose, and chlorine tended to be normal. IVT tigecycline + amikacin was discontinued, and the extraventricular drain was removed on hospital day 23. IV antibiotics continued to be used because on hospital day 19, the patient’s left eyebrow arch skin was torn with purulent secretions. On hospital day 22, the secretion culture implied an multidrug-resistant *K. pneumoniae* infection that was sensitive to tigecycline, amikacin, and imipenem. In addition, the subcutaneous secretions were repeatedly washed with normal saline every day, but the patient’s body temperature did not significantly improve (approximately 37.5°C), and the secretions continued to increase. On hospital day 24, according to the advice of the clinical pharmacist, the patient underwent debridement of the scalp infection and implant removal. The postoperative antibiotics were changed to imipenem and cilastatin sodium 2 g q8h IV + amikacin 0.8 g qd IV. Three days later, the skin wound healed well without exudation and a daily CSF examination showed no significant abnormalities. Unfortunately, the patient again reported increased sputum volume and elevated infection indicators (WBC, 21.44 × 10^9^/L; PCT, 1.46 ng/mL), which was suspected to be a new pneumonia infection. On hospital day 29, the sputum culture from hospital day 26 revealed a mixed infection of multidrug-resistant *K. pneumoniae* and *P. aeruginosa*. Based on the results of the drug sensitivity test, amikacin was adjusted to ciprofloxacin 0.4 g q8h IV. Seven days later, the patient’s body temperature and infection indicators returned to normal levels. Meanwhile, 3 consecutive sputum cultures from hospital day 29 yielded negative results. Eventually, the patient’s intracranial, skin soft tissue, and lung infections were all cured. On hospital day 38, the patient was transferred to the general ward for functional recovery. No side effects were observed during the dual IVT antibiotic therapy. Continued 6-month-follow-up showed no recurrence and the patient was able to walk slowly on his own.

**Table 1:** Laboratory tests for CSF in the period of treatment.

Date	Hospital day 5	Hospital day 9	Hospital day 13	Hospital day 15	Hospital day 18	Hospital day 21	Hospital day 25	Hospital day 33	Hospital day 41	Hospital day 65
WBC(× 106/L)	2	885	836	1078	185	68	30	29	61	77
LMR (%)	50	97.7	77.9	91.4	91.9	82.3	23.4	17.2	22.9	5.2
Glucose (mmol/L)	4.96	0.17	4.00	5.11	4.60	3.33	3.34	2.97	3.03	3.12
Protein (mg/dL)	18.1	281.7	90.9	134.6	60.1	102.8	64.8	89.1	103.2	104.0

**Table 2:** Antibiotics susceptibility tests for *Klebsiella pneumoniae*.

	MIC (mg/L): susceptibility interpretation*		
Drug	Hospital day 10; source: sputum	Hospital day 11; source: CSF	Hospital day 18; source: CSF
Amoxicillin/Clavulanate	≥32: Resistant	≥32: Resistant	≥32: Resistant
Piperacillin/Tazobactam	≥128: Resistant	≥128: Resistant	≥128: Resistant
Cefuroxime	≥64: Resistant	≥64: Resistant	≥64: Resistant
Cefoxitin	≥64: Resistant	≥64: Resistant	≥64: Resistant
Ceftazidime	≥64: Resistant	≥64: Resistant	≥64: Resistant
Ceftriaxone	≥64: Resistant	≥64: Resistant	≥64: Resistant
Cefoperazone/Sulbactam	≥64: Resistant	≥64: Resistant	≥64: Resistant
Cefepime	≥32: Resistant	≥32: Resistant	≥32: Resistant
Ertapenem	≥8: Resistant	≥8: Resistant	≥8: Resistant
Imipenem	≥16: Resistant	≥16: Resistant	≥16: Resistant
Amikacin	≤2: Susceptible	≤2: Susceptible	≤2: Susceptible
Gentamicin	17mm: Susceptible (K-B)	16mm: Susceptible (K-B)	20mm: Susceptible (K-B)
Levofloxacin	≥8: Resistant	≥8: Resistant	≥8: Resistant
Tigecycline	2: Susceptible	2: Susceptible	2: Susceptible
Trimethoprim/Sulfonamide	≥320: Resistant	≥320: Resistant	≥320: Resistant
Ceftazidime/avibactam	17mm: Resistant (K-B)	17mm: Resistant (K-B)	19mm: Resistant (K-B)

## 3. Discussion

In recent years, CNSIs caused by multidrug-resistant *A. baumannii* and *K. pneumoniae* after neurosurgery have been reported continuously, which have caused serious global health problems. Due to the poor permeability of blood-brain barrier and lack of sensitivity of most drugs, IVT antibiotics therapy for intracranial infections becomes a hot topic. The 2017 Infectious Diseases Society of America’s Clinical Practice Guidelines for Healthcare-associated Ventriculitis and Meningitis support the use of IVT antimicrobials for the treatment of difficult-to-eradicate or multidrug-resistant CSF infections.^[5]^ Nowadays, a series of clinical studies revealed that IVT colistin can successfully treat patients with extremely drug-resistant *A. baumannii* meningitis/ventriculitis.^[5,6]^Nevertheless, the neurotoxicity^[7]^of intraventricular injection of colistin (as high as 21.7%) and the heterogeneous resistance^[8]^ to colistin monotherapy have restricted its further application.

Tigecycline is the first novel semisynthetic antimicrobial agent of glycylclines, which blocks t-RNA from entering ribosome site A by reversibly binding to the bacterial ribosome 30S subunit, resulting in inhibition of bacterial protein synthesis and bacterial growth. In addition, tegecycline effectively avoids the 2 genetic mechanisms of tetracycline resistance (efflux and ribosomal protection), by adding a glycyclamide moiety to the 9-position of minocycline, further expanding the broad-spectrum antibacterial activity of tetracycline.^[9]^ In vitro, tigecycline showed strong antibacterial activity against a variety of drug-resistant bacteria, including CRKP.^[9,10]^ The 2020 CHINET data showed that tigecycline was 90.9% sensitive to 8858 CRKP strains. Unfortunately, tegecycline also has poor CSF penetration and has not been approved by the FDA for CNSIs. A study on the drug concentration distribution of tigecycline in different tissues after a single dose of 100 mg in healthy adults showed that the ratio of CSF to serum AUC was only 0.11.^[11]^This is similar to the results obtained by Munyeza et al^[12]^ to quantitatively determine the tigecycline content in rat brain tissue by liquid chromatography-tandem mass spectrometry (LC-MS/MS). A series of studies by Dandache et al^[13]^, Lengerke et al^[14]^, Ray et al,^[15]^and Pallotto et al^[16]^further showed that even in the presence of meningitis, the drug concentration of tigecycline in the CSF was still far lower than the MIC of pathogens. However, Mei et al^[17]^ reported a case of multidrug-resistant *K. pneumoniae* treated with IVT tigecycline, and the concentration of tigecycline in CSF was determined to be 17400–26400 ng/mL by LC-MS/MS, and the ratio of CSF to serum AUC calculated by the noncompartmental model was 48.9. Similarly, Wu et al^[18]^ reported a case of multidrug-resistant *K. pneumoniae* meningitis treated with 3 different doses of tigecycline (49 mg IV + 1mg IVT q12h, 45 mg IV + 5mg IVT q12h, and 40 mg IV + 10 mg IVT q12h), and the trough concentrations of tigecycline in CSF measured by LC-MS/MS were 0.313, 1.29, and 2.886 mg/L, respectively. The optimal trough concentration was obtained at the highest IVT tigecycline dosage, which was higher than the MIC (2 mg/L) of the pathogen. Soto-Hernández et al^[19]^ reported a case of IVT tigecycline for the treatment of multidrug-resistant *Klebsiella oxytogenogen* ventriculitis. Two hours after administration, the *C*_max_ of tigecycline in CSF was 178.9–310.1 mg/L by liquid chromatography-ultraviolet detection. Even after 6 hours, the drug concentration of tigecycline in CSF can still reach 35.4–41.3 mg/L, which is 15–20 times higher than the MIC (2 mg/L) of the pathogen. All 3 patients with CNSIs were cured without adverse reactions. Based on the current research, IVT antibiotics can significantly increase the drug concentration in the CSF, which is more conducive to the control of CNSIs.

The clinical efficacy of tigecycline monotherapy is not optimal for CRKP. To improve bacterial killing and inhibit the emergence of resistant strains, antibiotic combination therapy is recommended for the treatment of CRKP infections.^[20]^ However, the optimal combination of antibiotics for the treatment of CRKP infection is still unknown, and the common combination regimen is tigecycline + colistin, tigecycline + aminoglycoside or tigecycline + carbapenes (MIC ≤ 8mg/L). At present, the treatment of CNSIs caused by CRKP is mainly based on intraventricular injection of an antibiotic combined with one or more other systemic intravenous antibiotics,^[18,21–23]^ whereas there are few case reports of intraventricular injection of 2 antibiotics. Tsolaki et al^[24]^ reported 3 cases of CNSIs caused by extremely drug-resistant bacteria (one case was CRKP) successfully treated with combined IVT colistin and tigecycline, after the initial regimen of colistin administered alone through both IVT and IV routes had failed. The report shows that compared with IVT single colistin, combined administration is conducive to rapid microbial clearance and clinical improvement. Similarly, Nevrekar et al^[25]^ also reported the case of a patient with CRKP ventriculitis who underwent microbiological eradication with combination IVT antimicrobial therapy consisting of colistin and gentamicin plus systemic antimicrobials. In addition, a series of in vitro and in vivo synergistic tests^[26–28]^ of CRKP showed that tigecycline combined with aminoglycosides had good synergistic effects. Synergistic experiments by Ni et al^[29,30]^further showed that compared with the colistin-tigecycline and colistin-aminoglycosides combinations, the tigecycline-aminoglycosides combination could effectively suppress the resistance selection in *K. pneumoniae.*

Based on the pharmacokinetics of tigecycline in CSF and in vivo and in vitro synergies of tigecycline on CRKP, the patient had a homologous CRKP infection of the lung and central nervous system. The patient was administered IV plus IVT tigecycline-amikacin antiinfective therapy for the first time. However, there is no uniform standard dose of IVT tigecycline. In this case, we administered intraventricular tigecycline 3 mg q12h because in previous reports, pathogens in the CSF were difficult to remove when low doses were administered.^[24,31]^ Based on a systematic review of intraventricular aminoglycoside drugs, intraventricular amikacin 30 mg daily was administered.^[32]^ Ultimately, the patient was treated with dual IVT combined with IV administration, the pathogenic bacteria in the CSF were eradicated after 7 days, and the CNSIs were completely cured after 14 days.

In conclusion, a man with CNSIs caused by CRKP was successfully treated with intravenous plus intraventricular tegacycline-amikacin. Therefore, intravenous plus intraventricular tigecycline-amikacin seems to be a safe and effective treatment for CNSIs. However, this report is only one case, and multicenter randomized studies are needed to demonstrate the efficacy and safety of dual intraventricular administration in patients.

## Author contributions

Conceptualization: Jiyao Li.

Data curation: Jiyao Li, Yiguo Liu, Hongyan Wang.

Formal analysis: Jiyao Li.

Investigation: Jiyao Li, Guangtao Wu, Yiguo Liu, Hongyan Wang.

Project administration: Jiyao Li, Xiaoyan Xu.

Supervision: Xiaoyan Xu.

Writing–original draft: Jiyao Li.

Writing–review & editing: Jiyao Li, Xiaoyan Xu.

## Footnotes

* Report time of the drug sensitivity test.

## References

[1] Karvouniaris M Brotis A Tsiakos K. Current perspectives on the diagnosis and management of healthcare-associated ventriculitis and meningitis. Infection and Drug Resistance 2022;15:697–721. 35250284 10.2147/IDR.S326456 PMC8896765

[2] Lee C-R Lee JH Park KS. Global dissemination of carbapenemase-producing klebsiella pneumoniae: epidemiology, genetic context, treatment options, and detection methods. Front Microbiol. 2016;7:895. 27379038 10.3389/fmicb.2016.00895 PMC4904035

[3] Fang YQ Zhan RC Jia W. A case report of intraventricular tigecycline therapy for intracranial infection with extremely drug resistant Acinetobacter baumannii. Medicine (Baltim). 2017;96:e7703. 10.1097/MD.0000000000007703 PMC5626159 28767605

[4] Tunkel AR Hasbun R Bhimraj A. 2017 Infectious Diseases Society of America’s clinical practice guidelines for healthcare-associated ventriculitis and meningitis. Clin Infect Dis. 2017;64:e34–65. 28203777 10.1093/cid/ciw861 PMC5848239

[5] De Bonis P Lofrese G Scoppettuolo G. Intraventricular versus intravenous colistin for the treatment of extensively drug resistant Acinetobacter baumannii meningitis. Eur J Neurol. 2016;23:6–75. 10.1111/ene.12789 26228051

[6] Yu XB Huang YY Zhang XS. Intraventricular colistin sulphate as a last resort therapy in a patient with multidrug-resistant Acinetobacter baumannii induced post-neurosurgical ventriculitis. Br J Clin Pharmacol. 2022;88:3490–494. 35060164 10.1111/bcp.15238

[7] Karaiskos I Galani L Baziaka F. Intraventricular and intrathecal colistin as the last therapeutic resort for the treatment of multidrugresistant and extensively drug-resistant Acinetobacter baumannii ventriculitis and meningitis: a literature review. Int J Antimicrob Agents. 2013;41:499–508. 23507414 10.1016/j.ijantimicag.2013.02.006

[8] Band VI Satola SW Smith RD. Colistin heteroresistance is largely undetected among carbapenem-resistant enterobacterales in the United States. mBio 2021;12:e02881–20. 33500343 10.1128/mBio.02881-20 PMC7858057

[9] Yaghoubi S Zekiy AO Krutova M. Tigecycline antibacterial activity, clinical effectiveness, and mechanisms and epidemiology of resistance: narrative review. Eur J Clin Microbiol Infect Dis. 2021;5:1–20. 10.1007/s10096-020-04121-1 PMC7785128 33403565

[10] Karlsson M Lutgring JD Ansari U. Molecular characterization of carbapenem-resistant enterobacterales collected in the United States. Microb Drug Resist. 2022;28:389–97. 35172110 10.1089/mdr.2021.0106

[11] Rodvold KA Gotfried MH Cwik M. Serum, tissue and body fluid concentrations of tigecycline after a single 100 mg dose. J Antimicrob Chemother. 2006;58:1221–9. 17012300 10.1093/jac/dkl403

[12] Munyeza CF Shobo A Baijnath S. Development and validation of a liquid chromatography-tandem mass spectrometry (LC-MS/MS) method for the quantification of tigecycline in rat brain tissues. Biomed Chromatogr. 2016;30:837–45. 26378888 10.1002/bmc.3616

[13] Dandache P NicolauI DP Sakoulas G. Tigecycline for the treatment of multidrug-resistant klebsiella pneumoniae meningitis. Infectious Diseases in Clinical Practice. 2009;17:66–8.

[14] Lengerke C Haap M Mayer F. Low tigecycline concentrations in the cerebrospinal fluid of a neutropenic patient with inflamed meninges. Antimicrob Agents Chemother. 2011;55:449–50. 20937792 10.1128/AAC.00635-10 PMC3019631

[15] Ray L Levasseur K Nicolau DP. Cerebral spinal fluid penetration of tigecycline in a patient with Acinetobacter baumannii cerebritis. Ann Pharmacother;2010, 44:582–6. 20179255 10.1345/aph.1M480

[16] Pallotto C Fiorio M D’Avolio A. Cerebrospinal fluid penetration of tigecycline. Scand J Infect Dis. 2014;46:69–72. 24131423 10.3109/00365548.2013.837957

[17] Mei S Luo X Li X. Development and validation of an LC-MS/MS method for the determination of tigecycline in human plasma and cerebrospinal fluid and its application to a pharmacokinetic study. Biomed Chromatogr. 2016;30:1992–2002. 27245381 10.1002/bmc.3776

[18] Wu Y Chen K Zhao J. Intraventricular administration of tigecycline for the treatment of multidrug-resistant bacterial meningitis after craniotomy: a case report. J Chemother. 2017;30:49–52. 28614982 10.1080/1120009X.2017.1338846

[19] Soto-Hernández JL Soto-Ramírez A Pérez-neri I. Multidrug-resistant Klebsiella oxytoca ventriculitis, successfully treated with intraventricular tigecycline: A case report. Clin Neurol Neurosurg. 2020;188:105592. 31760254 10.1016/j.clineuro.2019.105592

[20] Ni WT Han YL Liu J. Tigecycline treatment for carbapenem-resistant enterobacteriaceae infections, a systematic review and meta-analysis. Medicine (Baltim). 2016;95:e3126. 10.1097/MD.0000000000003126 PMC4839946 26986165

[21] Gofman N To K Whitman M. Successful treatment of ventriculitis caused by Pseudomonas aeruginosa and carbapenem-resistant Klebsiella pneumoniae with i.v. ceftazidime–avibactam and intrathecal amikacin. Am J Health Syst Pharm. 2018;75:953–7. 29941534 10.2146/ajhp170632

[22] Curebal B Dalgic N Bayraktar B. Intraventricular tigecycline for the treatment of shunt infection: a case in pediatrics. J Neurosurg Pediatr. 2018;23:247–50. 30497136 10.3171/2018.9.PEDS18470

[23] Emiroglu M Alkan G Turk Dagi H. Tigecycline therapy in an infant for ventriculoperitoneal shunt meningitis. Pediatrics. 2017;139:e20160963. 27974589 10.1542/peds.2016-0963

[24] Tsolaki V Karvouniaris M Manoulakas E. Intraventricular CNS treatment with Colistin-Tigecycline combination: a case series. J Crit Care. 2018;47:338–41. 30097260 10.1016/j.jcrc.2018.07.025

[25] Nevrekar S Cunningham KC Greathouse KM. Dual intraventricular plus systemic antibiotic therapy for the treatment of klebsiella pneumoniae carbapenemase-producing klebsiella pneumoniae ventriculitis. Ann Pharmacother. 2014;48:274–8. 24259634 10.1177/1060028013510487

[26] Demirlenk YM Gücer LS Uçku D. A meta-analysis for the role of aminoglycosides and tigecyclines in combined regimens against colistin- and carbapenem-resistant Klebsiella pneumoniae bloodstream infections. Eur J Clin Microbiol Infect Dis. 2022;41:761–69. 35303195 10.1007/s10096-022-04429-0

[27] Tang H-J Lai C-C Chen C-C. Colistin-sparing regimens against Klebsiella pneumoniae carbapenemase-producing K. pneumoniae isolates: Combination of tigecycline or doxycycline and gentamicin or amikacin. J Microbiol Immunol Infect. 2019;52:273–81. 27133391 10.1016/j.jmii.2016.03.003

[28] Nulsopapon P Nasomsong W Pongchaidecha M. The synergistic activity and optimizing doses of tigecycline in combination with aminoglycosides against clinical carbapenem-resistant klebsiella pneumoniae isolates. Antibiotics (Basel). 2021;10:736. 34204561 10.3390/antibiotics10060736 PMC8234075

[29] Ni WT Wei CQ Zhou CF. Tigecycline-amikacin combination effectively suppresses the selection of resistance in clinical isolates of KPC-producing klebsiella pneumoniae. Front Microbiol. 2016;7:1304. 27594855 10.3389/fmicb.2016.01304 PMC4990548

[30] Ni WT Yang DQ Guan J. In vitro and in vivo synergistic effects of tigecycline combined with aminoglycosides on carbapenem-resistant Klebsiella pneumoniae. J Antimicrob Chemother. 2021;76:2097–105. 33860309 10.1093/jac/dkab122

[31] Long WY Yuan J Liu J. Multidrug resistant brain abscess due to acinetobacter baumannii ventriculitis cleared by intraventricular and intravenous tigecycline therapy: a case report and review of literature. Front Neurol. 2018;9:518. 30026723 10.3389/fneur.2018.00518 PMC6042469

[32] LeBras M Chow I Mabasa VH. Systematic review of efficacy, pharmacokinetics, and administration of intraventricular aminoglycosides in adults. Neurocrit Care. 2016;25:492–507. 27043949 10.1007/s12028-016-0269-3

